# Depression, Functional Dependence, and Oral Health: Evidence from a Clinical Study of Older Spanish Adults

**DOI:** 10.3390/diagnostics15222934

**Published:** 2025-11-20

**Authors:** Carmen Esperanza Abregú-Flores, Pedro Luis Ruiz-Sáenz, María Andrés-Veiga, Fernando Fernández-Cáliz, Cristina Meniz-García, Natalia Martínez-Rodríguez

**Affiliations:** 1 Department of Dental Clinical Specialties, Faculty of Dentistry, Complutense University of Madrid, 28040 Madrid, Spain; cabregu@ucm.es (C.E.A.-F.); fernanfe@ucm.es (F.F.-C.); nataliamartinez@ucm.es (N.M.-R.); 2 Department of Odontology, Central Hospital of the Red Cross of Madrid, 28003 Madrid, Spain; drruizsaenz@gmail.com; 3 Faculty of Health Sciences, Alfonso X University (UAX), 28691 Madrid, Spain; mandrvei@uax.es

**Keywords:** depression, oral health, geriatric dentistry, functional dependence, older adults

## Abstract

**Background**: Depression is a prevalent mental health condition among older adults and may be linked to multiple systemic and functional factors. Emerging evidence suggests a bidirectional relationship between depressive symptoms and poor oral health, but data from European populations remain scarce. **Methods**: A cross-sectional study was conducted in 181 community-dwelling adults aged over 66 years in Madrid, Spain. Depressive symptoms were assessed using the Geriatric Depression Scale (GDS-15), and functional dependence using the Barthel Index. Oral examinations included the number of caries, root remnants, and gingivitis. Additional variables included the Body Mass Index (BMI), smoking and alcohol consumption, and medication use. Data were analysed using descriptive and non-parametric statistics. **Results**: Overall, 49.2% of participants exhibited depressive symptoms (34.8% moderate, 14.4% severe). Old age and excess weight were significantly associated with depression (*p* < 0.05). Depressed participants showed higher functional dependence scores on the Barthel Index (*p* < 0.001). Oral health indicators, including higher number of caries, root remnants, and gingivitis, were significantly worse among those with depression (*p* < 0.005). Oral hygiene practices such as toothbrushing were slightly more frequent in the non-depressed group, while oral irrigator use was low across all groups. **Conclusions**: Depressive symptoms in older Spanish adults were associated with poorer oral health, greater functional dependence, and certain clinical factors such as BMI and antidepressant use. These findings highlight the importance of integrated, multidisciplinary approaches to promote both mental and oral health in ageing populations. Longitudinal studies are warranted to clarify the causal pathways underlying these associations.

## 1. Introduction

Depression is a prevalent mental disorder among older adults worldwide, with substantial variability in prevalence across countries due to multifactorial influences, including age, gender, socioeconomic status, psychosocial stressors, and comorbid health conditions [1]. Depression rates have been reported at 10.3% in China [2], 6.4% in the United States [3], 10.7% in Brazil [4], 16% among older adults in Australia [5], and up to 50.4% in Ethiopia [6]. In Europe, a study conducted in 27 countries showed that the overall prevalence among individuals aged 65 and older was 35.1% in women and 21.5% in men, although it has declined over the past two decades, particularly in Southern Europe [7].

In parallel, oral diseases such as dental caries, periodontitis, tooth loss, and oral cancer affect approximately 3.5 billion people globally, accounting for nearly half of the world’s population [8]. Recent research has emphasised a possible bidirectional association between oral health and mental health: depression and loneliness are linked to increased risk of periodontal disease progression [9,10], while poor oral health—such as caries, edentulism, and tooth loss—can contribute to worsening depressive symptoms, especially among older adults [11]. This association could be explained by factors such as chronic pain, impaired speech and mastication, aesthetic concerns, and subsequent impacts on self-esteem and social interaction [12,13].

To facilitate the early detection of depression in older adults, tools such as the 15-item Geriatric Depression Scale (GDS) have been developed and validated, demonstrating high sensitivity (79–100%) and specificity (67–80%) [14,15]. Studies using this scale have further explored the relationship between oral health and depression. For example, Yamamoto et al. [16] reported that tooth loss and poor oral status were associated with depressive symptoms in a large sample of older Japanese adults. Wright et al. [17] found that oral health may contribute to depression among older Australian men, particularly factors such as chewing capacity and the presence of decayed teeth, although the direction of causality remains unclear.

Despite growing global interest, there is a scarcity of research examining this relationship in European older adult populations. This study aims to investigate the association between depressive symptoms, oral health status, and functional dependence in a community-dwelling sample of older adults in Spain. The null hypothesis was that depressive states or functional dependence in older adults do not influence oral health status. Additionally, the study explores the influence of covariates such as tobacco and alcohol use, Body Mass Index (BMI), and medication consumption.

## 2. Materials and Methods

### 2.1. Study Design

A cross-sectional prospective clinical study was conducted in an elderly population aged over 66 years. All participants were informed verbally and in writing about the study and gave their signed informed consent. The study adhered to the principles of the Declaration of Helsinki and received ethical approval from the Ethics Committee of Hospital Clínico San Carlos in Madrid (CI 22/120-E).

### 2.2. Participants

A total of 181 non-institutionalised patients of both sexes were prospectively and consecutively recruited from the Dental Service of the Central Red Cross Hospital of Madrid and the School of Dentistry at the Complutense University of Madrid. Before inclusion, all participants were confirmed to be cognitively competent through psychiatric and neurological evaluations, which included the Mini-Mental State Examination (MMSE) [18]. Patients with degenerative mental illnesses (e.g., Alzheimer’s disease) or terminal illnesses were excluded. MMSE scores between 27 and 30 indicated no cognitive impairment.

### 2.3. Questionnaire Data

The first questionnaire, administered by two researchers (P.L.R.-S and C.M.-G.), aimed to detect depressive symptoms using the short form of the GDS [14,15], which includes 15 items. Affirmative responses scored 0, and negative responses scored 1, resulting in a total score between 0 and 15 (Table 1). Participants were classified as follows: 0–5 = no depression, 6–10 = moderate depression, 11–15 = severe depression.

A second questionnaire, administered by two other researchers (C.E.A.-F and M.A.-V), evaluated functional capacity using the Barthel Index [19]. This index includes 10 items and gives a score between 0 (total dependence) to 100 (completely independent) (Table 2).

### 2.4. Smoking and Alcohol Consumption

Participants were asked about their smoking habits and alcohol consumption. Smokers were categorised as: (1) non-smoker; (2) <10 cigarettes/day; (3) 10–20 cigarettes/day; (4) >20 cigarettes/day; (5) former smoker [20].

Alcohol consumption was assessed using the Alcohol Use Disorders Identification Test–Short Form (AUDIT-C), adapted to Spanish. This questionnaire includes three items that assess drinking frequency, average consumption, and binge drinking. Responses follow a Likert scale from 0 (low consumption) to 4 (high consumption), with an additional option for former drinkers scored as 5 [21].

### 2.5. General Health Data

Participants were asked about their current medication use [22], specifically the use of antidepressants.

The BMI was calculated by dividing the weight in kilograms by the height in square metres [23]. The following categories were used:

- Severely underweight: BMI < 16.5 kg/m^2^
- Underweight: BMI 16.5–18.4 kg/m^2^
- Normal weight: BMI 18.5–24.9 kg/m^2^
- Overweight: BMI 25–29.9 kg/m^2^
- Obese: BMI ≥ 30 kg/m^2^. This category was further subdivided into: (1) Class I obesity: BMI 30–34.9 kg/m^2^; (2) Class II obesity: BMI 35–39.9 kg/m^2^; (3) Class III obesity: BMI ≥ 40 kg/m^2^ (also known as severe, extreme, or morbid obesity).

### 2.6. Oral Health Data

The oral examination was performed by two experienced dentists (C.E.A.-F. and P.L.R.-S.), who recorded the results in the clinical charts: the number of present teeth, root remnants, dental caries, root caries, and tooth mobility.

The Modified Gingival Index (MGI) [24] was categorised as: (0) Normal gingiva; (1) Mild inflammation—slight colour change, slight oedema, no bleeding on probing; (2) Moderate inflammation—redness, oedema, glazing, bleeding on probing; (3) Severe inflammation—marked redness and oedema, ulceration, tendency for spontaneous bleeding. Each gingival unit was scored from 0 to 3 by assessing the mesial, buccal, lingual/palatal, and distal surfaces.

Participants were also asked about daily toothbrushing frequency, recorded as: (0) none; (1) once; (2) twice; (3) three times per day. The use of oral irrigators was also recorded (yes/no) [25].

### 2.7. Statistical Analysis

Statistical analyses were performed using IBM SPSS Statistics for Windows (version 27.0, IBM Corp., Armonk, NY, USA). The inter-operator agreement for the oral health variables was assessed using Cohen’s kappa coefficient [26]. The level of agreement was determined according to the criteria of Landis and Koch, requiring values greater than 0.80 [27]. Descriptive statistics (frequencies and percentages) were calculated for all variables, both for the whole sample and for the subgroups.

The normality of the quantitative variables was assessed using the Kolmogorov–Smirnov and Shapiro–Wilk tests to determine data distribution. For the quantitative variables that did not follow a normal distribution, the non-parametric Kruskal–Wallis test was used to compare the groups. The qualitative variables were analysed using the chi-square test. Adjusted residuals were examined to identify which categories contributed significantly to the chi-square value; residuals outside the range of −2.0 to +2.0 indicated a significant difference between the observed and expected frequencies. A *p*-value < 0.05 was considered statistically significant.

## 3. Results

### 3.1. Sample Characteristics

**Figure 1:**
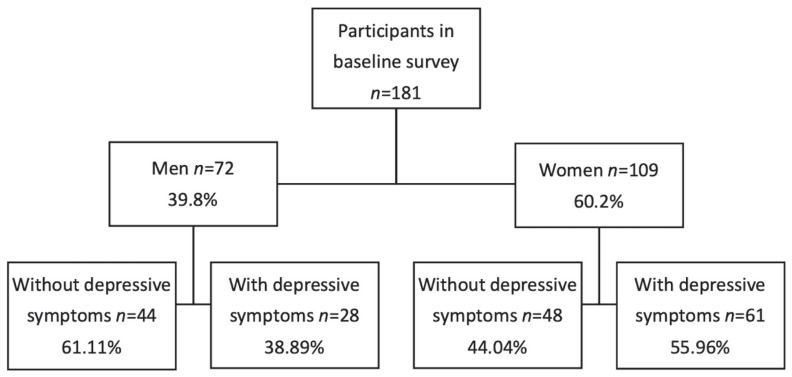
Description of the sample.

A total of 181 participants were included in the study, comprising 72 males (39.8%) and 109 females (60.2%), with females representing the majority of the sample (Figure 1).

### 3.2. GDS Questionnaire Results and Correlations with Other Variables

According to the GDS, Table 3 shows that among the 181 participants, 92 (50.8%) had no depression, 63 (34.8%) presented moderate depression, and 26 (14.4%) presented severe depression.

Table 4 displays the associations between different variables and the three GDS subgroups. Age appeared to be a significant factor, with older participants more frequently presenting depressive symptoms.

Regarding gender, although more than half of the women (56.0%) self-reported moderate to severe depression compared with men (38.9%), the severity of depression did not differ significantly between genders. Specifically, the proportion of women was higher in the moderate (40.4%) and severe (15.6%) depression groups compared with the non-depressed group (44.0%). Conversely, men represented a higher percentage in the non-depressed group (61.1%) than in both depressed groups (26.4% and 12.5%, respectively).

In terms of smoking habits, the majority of participants were non-smokers (34.80%) or former smokers (48.07%), accounting for a combined 82.87%. Comparison between moderate and severe depression groups and the non-depressed group showed very similar results (*p* = 0.191).

As for alcohol consumption, a total of 123 participants (67.95%) reported not having this habit. Eighty-three participants (45.86%) were abstainers, and 40 (22.09%) were former drinkers. When analysing both conditions together (abstainer and former drinker), a similar distribution was observed among the study groups: 63 non-depressed participants compared with 60 depressed participants (38 with moderate depression and 22 with severe depression). However, with increased alcohol intake, a pattern emerged: participants in alcohol consumption category 3 tended to be non-depressed (81.8%), while those in category 4 were more likely to be moderately (66.7%) or severely (22.2%) depressed (overall: 88.9%). Although this difference was statistically significant, the small number of cases in these subgroups suggests that results should be interpreted cautiously.

Body weight analysis revealed that 61.87% of participants were overweight or obese. Those who were overweight were more frequently found among the moderately depressed group (43.5%), whereas individuals with Class I obesity were significantly more frequent in the non-depressed group (70.7%).

Antidepressant use was significantly higher among participants with moderate depression (43.1%) compared to the non-depressed group (35.8%). Regarding general medication use, participants taking fewer than five medications were more common in the non-depressed group (60.3%) than in both moderately and severely depressed groups (31.8% and 7.9%, respectively). Reports of dry mouth were higher in the non-depressed group, although the difference was not statistically significant.

### 3.3. Barthel Index (Functional Dependence Status)

Table 5 summarises the functional capacity of participants based on the Barthel Index. Among participants without depression, more than half (56 individuals) were independent or had only slight dependence. Moderate dependence was observed in 28 individuals, and severe dependence in 8; there were no cases of total dependence. In contrast, among participants with depression, only 8 individuals (from the moderate depression group) were independent or slightly dependent. In the moderate and severe depression groups, most participants were classified as having severe or total dependence. These findings indicate a statistically significant relationship between depression severity and functional dependence (*p* < 0.001).

### 3.4. Oral Health Status

The inter-operator agreement, assessed using Cohen’s kappa coefficient, was 0.9, indicating an almost perfect level of agreement. As shown in Table 6, the most relevant finding was that the presence of root remnants was notably higher in severely depressed individuals, with a strong statistical significance (*p* < 0.001).

Table 7 shows the MGI results. Normal gingiva (no gingivitis) was more frequent in the non-depressed group, while moderate and severe gingivitis were significantly more common among moderately or severely depressed participants (*p* < 0.001).

Regarding toothbrushing frequency, results were generally similar between groups, except for those who reported brushing three times per day: this behaviour was more common in the non-depressed group (62.5%). As for oral irrigator use, the majority of participants answered “no use”, with no significant differences between groups.

## 4. Discussion

The scientific community has increasingly focused on identifying the risk factors for depression, including the role of oral health. It remains unclear whether poor oral health contributes to depression or whether depression leads to neglect of oral hygiene. Therefore, this study may contribute to the limited evidence from European populations, particularly in Spain, by exploring clinical characteristics, functional status, oral health, and lifestyle habits. The results obtained reject the null hypothesis proposed at the beginning.

Older age was significantly associated with higher levels of depressive symptoms, consistent with the findings of Assariparambil et al. [28], Hu et al. [29], and Sempértegui et al. [30]. While more women in our sample presented with depression, the gender difference was not statistically significant, though other studies have identified female gender as a predisposing factor [31,32,33].

Regarding lifestyle habits, no significant association was found between smoking and depression. This contrasts with studies by Kiran et al. [34], Bakhshaie et al. [35], and Liu et al. [36], which reported a clear link between smoking and depression severity. This discrepancy could stem from the low number of smokers in our sample or from cultural differences, as the cited studies were conducted in Indian, U.S., and Chinese populations.

Alcohol consumption presented a mixed picture. Although most participants were abstainers or former drinkers, higher levels of alcohol intake were associated with depression. These findings partially align with those of Verlinden et al. [37] and Cobb et al. [38], who observed a positive relationship between alcohol use and depression. In contrast, Scott et al. [39] and Gea et al. [40] found that moderate alcohol consumption may reduce depressive symptoms, while excessive intake increases risk.

BMI was another relevant factor. In line with Schrempft et al. [41], our study found that overweight individuals were more likely to experience depression. Similar findings were also reported by Bludau et al. [42], who, in a German population of 2568 patients, observed a statistically significant association with obesity. However, studies in Asian populations, such as those by Qiao et al. [43] and Hong & Hur [44], suggest that being underweight is more strongly associated with anxiety, again indicating the possible influence of the cultural context.

Medication use was widespread in our sample, and polypharmacy (defined as >5 drugs) was common. This is consistent with findings by Neumann-Podczaska et al. [45] and Al-Azayzih et al. [46]. Although polypharmacy was more prevalent among depressed patients in our study, the association was not statistically significant. However, other studies, including those by Eyigor et al. [47] and Chen et al. [48], found significant associations. Similarly, while some studies link polypharmacy to dry mouth [49], we did not observe significant differences in this symptom between groups.

A notable finding was the significant association between depression severity and functional dependence, as assessed by the Barthel Index. This is in line with previous research by Topsakal & Oğuz [50], Zhang & Yang [51], and Jin & Jing [52], who emphasise the importance of cognitive function and participation in daily activities to reduce depression.

In terms of oral health, we observed a clear association between depression and poor dental outcomes, including untreated caries, root remnants, and gingivitis. Kisely et al. [53] highlighted that poor oral health can predispose individuals to chronic physical and mental illnesses, leading to preventable hospitalizations for medical reasons. In the same vein, the findings of Wright et al. [17], Barbosa et al. [54], and Kunrath and Silva [55] support the idea that poor oral health is a contributing factor to depressive symptoms. This evidence strengthens the hypothesis of a bidirectional relationship between depression and oral health, as noted in earlier studies [9,10,11,56]. Therefore, as a preventive strategy, dental treatment for these patients is essential. Regular visits and proper prophylaxis can help prevent the development of periodontitis, an important step not only in tooth preservation but also in maintaining the patient’s overall health [57]. Avoiding tooth loss and its subsequent replacement with removable prostheses, when socioeconomic conditions do not allow other alternatives, could also help prevent the development of depressive symptoms. In this regard, Palomer et al. [58] found in a study of 2953 individuals that removable prostheses and phonatory difficulties are destabilising factors in patients suffering from depression.

Oral hygiene habits were also assessed. While overall brushing frequency was similar, brushing three times daily was more common among non-depressed participants. This differs from the findings of Cui et al. [59], who reported that brushing at least once daily reduced depression risk. The low use of oral irrigators, with no significant difference between groups, may reflect cultural factors in the Spanish population. However, their inclusion in daily hygiene routines could be beneficial, as suggested by Altalhi et al. [60] and Ren et al. [61].

Overall, this study confirms the complex, multidimensional relationship between mental and oral health in older adults. These findings support the need for integrated care approaches involving medical, psychological, and dental professionals.

This study has several limitations that should be acknowledged. First, its cross-sectional design does not allow for causal inferences between depression and oral health outcomes; longitudinal studies are required to determine the directionality of these associations. Second, the sample was recruited from a single urban area in Madrid, which may limit the generalisability of the findings to other Spanish or European populations. Third, although validated tools such as the GDS-15 and the Barthel Index were used, self-reported information on lifestyle habits (e.g., smoking, alcohol intake, and oral hygiene practices) could be subject to recall or social desirability bias. Additionally, the relatively low prevalence of some types of behaviour, such as current smoking and oral irrigator use, restricted our ability to explore these associations in depth. Finally, while we adjusted for several covariates, residual confounding due to unmeasured factors such as socioeconomic status or comorbidities cannot be excluded.

## 5. Conclusions

In this study of community-dwelling older adults in Spain, depressive symptoms were closely associated with poorer oral health outcomes, greater functional dependence, and certain clinical factors such as age, BMI, and antidepressant use. These findings confirm the multidimensional relationship between mental and oral health, emphasising the need for comprehensive geriatric care that integrates medical, psychological, and dental perspectives. Although oral hygiene habits appeared broadly similar across groups, a higher frequency of toothbrushing was observed among non-depressed participants, suggesting a potential protective role of self-care practices.

The results highlight the importance of early identification and management of depression in older adults as part of holistic health strategies. Future longitudinal studies are required to clarify the causal pathways underlying these associations and to evaluate the effectiveness of integrated interventions aimed at improving both mental well-being and oral health in ageing populations.

## Figures and Tables

**Table 1:** Items in the Geriatric Depression Scale (GDS-15).

Item	Question	Yes	No
1	Are you basically satisfied with your life?		
2	Do you continue to engage in your usual activities and interests?		
3	Do you feel that your life is full and happy?		
4	Do you find enjoyment in your daily activities?		
5	Are you generally in good spirits most of the time?		
6	Do you live your life without fear?		
7	Are you content during the day?		
8	Do you feel cared for or looked after?		
9	Do you prefer going out of your home or room rather than staying in?		
10	Is your memory satisfactory?		
11	Do you think it’s great to be alive?		
12	Do you feel useful as you are now?		
13	Do you feel full of energy?		
14	Do you believe your situation is not hopeless?		
15	Do you think most people are worse off than you?		
**Total score**			
0–5 = No depression 6–10 = Moderate depression 11–15 = Severe depression			

Yes, 0 points; No, 1 point.

**Table 2:** Items and scoring for the Barthel Index.

Function	Score and Description
Feeding	10 = Independent 5 = Needs help cutting food 0 = Dependent
Bathing	5 = Independent 0 = Dependent
Grooming	5 = Independent (face washing, hair, teeth, shaving) 0 = Dependent
Dressing	10 = Independent 5 = Needs help 0 = Dependent
Bowel Control	10 = Continent 5 = Occasional accident or needs help 0 = Incontinent
Bladder Control	10 = Continent 5 = Occasional accident or needs help 0 = Incontinent
Toilet Use	10 = Independent 5 = Needs some assistance 0 = Dependent
Transfers (bed to chair)	15 = Independent 10 = Minimal help 5 = Major help 0 = Dependent
Mobility (walking/wheelchair)	15 = Independent walking 10 = Needs supervision/help 5 = Independent in wheelchair 0 = Dependent
Stair Climbing	10 = Independent 5 = Needs help 0 = Dependent
**Levels of Dependence**	
100 = Independent 91–99 = Slight dependence 61–90 = Moderate dependence 21–60 = Severe dependence 0–20 = Total dependence	

**Table 3:** Distribution of patients according to the Geriatric Depression Scale (GDS).

GDS	No. Cases (*n* = 181)	Percentage
No depression	92	50.83%
Moderate depression	63	34.81%
Severe depression	26	14.36%

**Table 4:** Relation between the study groups and age, gender, smoking habit, alcohol consumption, Body Mass Index, antidepressant use, medication use, and dry mouth.

	No Depression (*n* = 92)	Moderate Depression (*n* = 63)	Severe Depression (*n* = 26)	*p* Value
**Average age (years)**	76.22 ± 9.41	83.40 ± 8.50	83.92 ± 7.29	<0.001 *
**Gender**				
Women (*n* = 109)	48 (44.0%)	44 (40.4%)	17 (15.6%)	0.074 **
Men (*n* = 72)	44 (61.1%)	19 (26.4%)	9 (12.5%)	
**Smoking habit**				
Non-smoker (*n* = 63)	28 (44.5%)	22 (34.9%)	13 (20.6%)	0.191 **
<10 cigarettes/day (*n* = 16)	7 (43.75%)	9 (56.25%)	0 (0.0%)	
10–20 cigarettes/day (*n* = 5)	3 (60.0%)	2 (40.0%)	0 (0.0%)	
>20 cigarettes/day (*n* = 10)	5 (50.0%)	5 (50.0%)	0 (0.0%)	
Former smoker (*n* = 87)	49 (56.3%)	25 (28.7%)	13 (15.0%)	
**Alcohol consumption**				
(0) Teetotaller (*n* = 83)	40 (48.2%)	25 (30.1%)	18 (21.7%) (+)	0.012 **
(1) Man = 30 g/day + Woman = 20 g/day (*n* = 18)	6 (33.3%)	11 (61.1%) (+)	1 (5.6%)	
(2) Man = 30–50 g/day + Woman = 20–32 g/day (*n* = 20)	13 (65.0%)	6 (30.0%)	1 (5.0%)	
(3) Man = 51–120 g/day + Woman = 33–80 g/day (*n* = 11)	9 (81.8%) (+)	2 (18.2%)	0 (0.0%)	
(4) Man >120 g/day + Woman >80 g/day (*n* = 9)	1 (11.1%) (−)	6 (66.7%) (+)	2 (22.2%)	
(5) Former drinker (*n* = 40)	23 (57.5%)	13 (32.5%)	4 (10.0%)	
**BMI**				
Severe underweight (*n* = 0)	0 (0.0%)	0 (0.0%)	0 (0.0%)	0.016 **
Underweight (*n* = 1)	0 (0.0%)	1 (100.0%)	0 (0.0%)	
Normal weight (*n* = 68)	38 (55.9%)	22 (32.3%)	8 (11.8%)	
Overweight (*n* = 46)	14 (30.4%) (−)	20 (43.5%)	12 (26.1%) (+)	
Class I obesity (*n* = 41)	29 (70.7%) (+)	8 (19.5%) (−)	4 (9.8%)	
Class II obesity (*n* = 12)	6 (50.0%)	6 (50.0%)	0 (0.0%)	
Class III obesity (*n* = 13)	5 (38.5%)	6 (46.1%)	2 (15.4%)	
**Antidepressant use**				
No (*n* = 58)	48 (82.8%) (+)	10 (17.2%) (−)	0 (0.0%) (−)	<0.001 **
Yes (*n* = 123)	44 (35.8%) (−)	53 (43.1%) (+)	26 (21.1%) (+)	
**Use of other medications**				
<5 medications (*n* = 63)	38 (60.3%)	20 (31.8%)	5 (7.9%)	0.093 **
≥5 medications (*n* = 118)	54 (45.8%)	43 (36.4%)	21 (17.8%)	
**Dry mouth sensation**				
No (*n* = 118)	60 (50.8%)	42 (35.6%)	16 (13.6%)	0.899 **
Yes (*n* = 63)	32 (50.8%)	21 (33.3%)	10 (15.9%)	

BMI, Body Mass Index; (−), adjusted residual values below −2; (+), adjusted residual values above +2; *, Kruskal–Wallis test; **, chi-square test.

**Table 5:** Relation between the study groups and the Barthel Index.

	No Depression (*n* = 92)	Moderate Depression (*n* = 63)	Severe Depression (*n* = 26)	*p* Value
**Total dependency** **Severe dependency** **Moderate dependency** **Low dependency** **Independent**	0 (0.0%) (−) 8 (20.5%) (−) 28 (44.4%) 9 (100.0%) (+) 47 (85.5%) (+)	14 (50.0%) 26 (66.7%) (+) 15 (34.9%) 0 (0.0%) (−) 8 (14.5%) (−)	14 (50.0%) (+) 5 (12.8%) 7 (20.6%) 0 (0.0%) 0 (0.0%) (−)	<0.001 **
**Average Barthel Index score and SD**	87.77 ± 18.29	54.44 ± 29.92	28.85 ± 28.99	<0.001 *

SD, standard deviation; *, Kruskal–Wallis test; **, chi-square test.

**Table 6:** Distribution and relations of the number of cavities, root caries and radicular remains.

	No Depression (*n* = 92)	Moderate Depression (*n* = 63)	Severe Depression (*n* = 26)	*p* Value *
	Mean/SD			
**Number of cavities**	3.13 ± 2.09	2.56 ± 2.76	1.85 ± 1.46	0.005
**Root caries**	2.21 ± 1.74	1.89 ± 2.11	2.19 ± 1.69	0.271
**Radicular remains**	1.42 ± 1.87	3.51 ± 3.83	6.69 ± 6.32	<0.001

*, Kruskal–Wallis test.

**Table 7:** Distribution and relations of gingivitis, brushing frequency, and use of water jet.

	No Depression (*n* = 92)	Moderate Depression (*n* = 63)	Severe Depression (*n* = 26)	*p* Value **
**MGI/Gingivitis**				
Absent-normal Mild Moderate Severe	19 (86.4%) (+) 42 (85.7%) (+) 28 (39.4%) (−) 3 (7.7%) (−)	1 (4.5%) (−) 7 (14.3%) (−) 36 (50.7%) (+) 19 (48.7%) (+)	2 (9.1%) 0 (0.0%) (−) 7 (9.9%) 17 (43.6%) (+)	<0.001
**Brushing frequency**				
None Once Twice 3 times	17 (41.5%) 23 (46.0%) 17 (50.0%) 35 (62.5%)	13 (31.7%) 19 (38.0%) 12 (35.3%) 19 (33.9%)	11 (26.8%) 8 (16.0%) 5 (14.7%) 2 (3.6%)	0.067
**Use of water jet**				
No Yes	74 (49.0%) 18 (60.0%)	52 (34.4%) 11 (36.7%)	25 (16.6%) 1 (3.3%)	0.159

**, chi-square test; (−), adjusted residual values below −2; (+), adjusted residual values above +2.

## Data Availability

The original contributions presented in this study are included in the article. Further inquiries can be directed to the corresponding author.

## Abbreviations

The following abbreviations are used in this manuscript:

GDS	Geriatric Depression Scale
BMI	Body Mass Index
MMSE	Mini-Mental State Examination
AUDIT-C	Alcohol Use Disorders Identification Test–Short Form
MGI	Modified Gingival Index
